# Warkany Syndrome: A Rare Case Report

**DOI:** 10.1155/2011/437101

**Published:** 2011-10-18

**Authors:** Amit Agrawal, Rashmi Agrawal

**Affiliations:** Department of Pediatrics, Chirayu Medical College & Hospital, Near Bairagarh, Bhopal 462030, India

## Abstract

Warkany syndrome 2 or Trisomy 8 mosaicism (T8M) is a well-described, but very rare, chromosomal abnormality. The phenotype is extremely variable ranging from normal individual to severe malformation syndrome and because of this variability, this condition often goes undiagnosed. We report trisomy 8 mosaicism (T8M) in a 3-year-old boy evaluated for facial dysmorphism and delayed development.

## 1. Introduction

Warkany syndrome 2 or Trisomy 8 mosaicism (T8M) is a well-described chromosomal abnormality with estimated frequency of about 1 : 25000 to 1 : 50000 births [1]. Complete trisomy 8 is usually lethal which occurs in 0.8% of spontaneous pregnancy losses but T8M has extremely variable phenotype ranging from normal individual to severe degree of malformations [2]. The phenotype includes an abnormal facies, reduced joint mobility, various vertebral and costal anomalies, eye anomalies, camptodactyly and deep plantar, and palmar creases. Deep plantar creases are highly characteristics of trisomy 8 mosaicism. We report a case of a 3-year-old male child brought for delayed development and abnormal facial features. His karyotype showed trisomy 8 mosaicism.

## 2. Case Report

**Figure 1:**
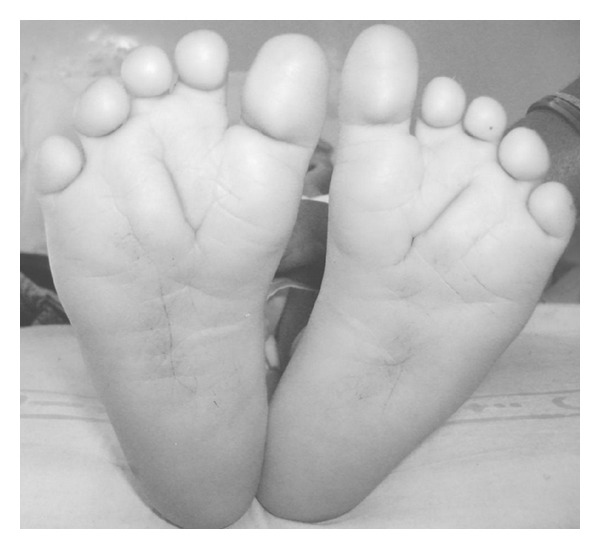
Characteristic deep plantar creases.

A 3-year-old male child born of nonconsanguineous marriage to a 28-year-old mother and a 32-year-old father by caesarean section was referred for evaluation for delayed development and abnormal facial features. There was a history of three spontaneous abortions. One pregnancy was terminated in late second trimester of gestation as the fetus was found to have multiple congenital anomalies on antenatal ultrasound. Exact cause of abortions could not be determined as chromosomal analysis of fetuses was not done, although TORCH screening of mother was negative. Antenatal, natal, and immediate postnatal history of the child described was not significant. After the age of three months, the parents noticed a lag in development along with abnormal facial features. Developmental assessment at the time of presentation revealed global delay. The child achieved neck holding at 12 months, sitting at 18 months and independent standing at 24 months of age. He had jargon speech with only few intelligible words. He was not able to feed himself. His weight was 11.0 kilograms (less than 5th percentile for age), height 75 cm (less than 5th percentile for age), and head circumference was 47 cm. Dysmorphic facial features included prominent forehead, large prominent ears with prominent antihelices, deep-set eyes, hypertelorism, bilateral corneal opacities, broad nose, thick everted lips, and high-arched palate. Orthopedic anomalies included camptodactyly of second through fifth fingers, mild scoliosis, short metatarsals, and deep plantar and deep palmar creases (Figure 1). X-ray spine showed mild scoliosis and posterior open neural arch defect in upper dorsal vertebra. Abdominal ultrasound, echocardiography, and cranial neuroimaging did not reveal any congenital anomaly of internal organs. 

**Figure 2:**
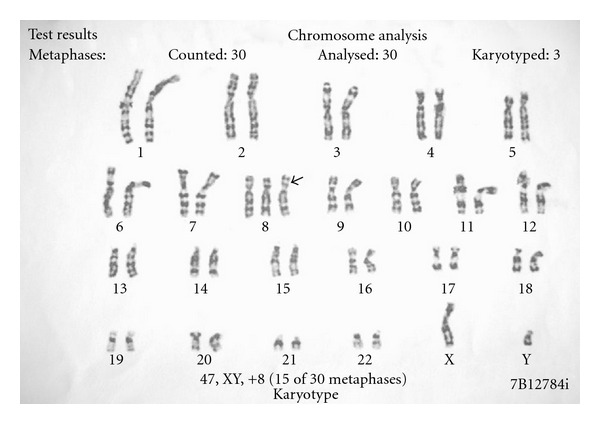
Karyotyping showing trisomy 8.

In view of presence of facial dysmorphism, musculoskeletal abnormalities, developmental delay, and history of recurrent loss of pregnancies, we got his karyotype done on peripheral lymphocytes, which revealed trisomy 8 mosaicism (47, XY, 6 + 8 [15]/46, XY [15]) (Figure 2). Chromosomal analysis of parents showed normal karyotype.

## 3. Discussion

Warkany syndrome 2 or Trisomy 8 mosaicism (T8M) is an autosomal abnormality with extremely variable phenotypic and cytogenetic expression [1, 2]. The estimated frequency is about 1 : 25000 to 1 : 50000 births, with a strong male predilection (M : F = 5 : 1) [1]. 

Grouchy first recognized a patient with trisomy 8 mosaicism in 1971. There is no significant difference in phenotype between the so-called pure trisomy 8 and trisomy 8 mosaicism. Both confined and generalized mosaicisms for trisomy 8 are, in majority of cases, due to mitotic nondisjunction during early zygotic development, whereas most cases of trisomy 8 observed in miscarriages are due to maternal meiotic errors. The phenotype includes prominent forehead, deep set eyes, and hypertelorism with broad nasal root, thick everted lips, prominent ears, camptodactyly of second through fifth fingers and toes, and deep plantar and palmar creases. Deep plantar creases are highly characteristic of this condition. Mental retardation is frequent and varies from mild to severe although some have normal intelligence. 

Besides these important clinical features, patients with T8M are at increased risk of developing leukemia and myelodysplastic syndrome, which may be due to altered stromal cell function leading to progenitor cell proliferation and expansion [3]. Occasional patient may have agenesis of corpus callosum. About 25% of patients may have congenital heart diseases (e.g., cardiomyopathy, septal defects, and great vessel anomalies). Our case had a number of characteristic clinical features associated with T8M patients, which have been previously reported in the literature and are presented in Table 1 [3, 4]. 

Renal malformations like hydronephrosis, ureteral reflux, and cryptorchidism are probably present in at least every second case. Partial extrahepatic biliary atresia may be present in association with T8M. Some authors have suggested that periconceptional toxoplasma infection may play a role in occurrence of such association [4]. Though antenatal diagnosis is possible using chorionic villous sampling (CVS), the results are not always confirmatory, because T8M found in CVS mostly represents confined placental mosaicism (CPM) sparing the fetus [5]. Maternal serum alpha-fetoprotein (MSAFP) has been reported to be markedly elevated in association with T8M [6]. Follow-up investigations including amniocentesis and especially fetal blood sampling are required for prenatal diagnosis of T8M. Unfortunately, both a normal amniocentesis and normal fetal blood sampling results cannot completely rule out chances of fetal T8M, and the detection of any abnormality on antenatal USG indicates that fetus may be affected. Clinical diagnosis of T8M is difficult because of subtle abnormality associated, and after-birth diagnosis is confirmed by performing karyotyping on peripheral blood cells and skin fibroblast culture. As the abnormal cell line tends to disappear from lymphocytes with age, fibroblast culture may be required in older patients to confirm the diagnosis [1]. 

In conclusion, chromosomal abnormality should be considered in children presenting with history of developmental delay and dysmorphic features.

## Figures and Tables

**Table 1:** 

****	Literature	Our patient
Short stature	+	+
Prominent forehead	+	+
Low posterior hairline	+	+
Plump nose with broad nose	+	+
Prominent nares	+	+
Prominent and deformed ears	+	+
Deep set eyes	+	+
Strabismus	+	−
Thick-everted lower lips	+	+
High-arched palate	+	+
Cleft palate	+	−
Camptodactyly of fingers	+	+
Deep palmar and plantar furrows	+	+
Pectus excavatum	+	−
Widely spaced nipples	+	+
Vertebral anomalies	+	+
Costal anomalies	+	−
Congenital heart disease	+	−
Urogenital anomalies	+	−
Agenesis of corpus callosum	+	−
